# From Congenital Torticollis to Leigh Syndrome: A Case Report of Diagnostic Evolution in an Infant

**DOI:** 10.3390/children12111522

**Published:** 2025-11-10

**Authors:** Minsoo Jeon, Shin-seung Yang, Sera Lee, Ja Young Choi

**Affiliations:** 1 Department of Rehabilitation Medicine, Deajeon Sejong Chungnam Nexon Sponsored Public Children’s Rehabilitation Hospital, Daejeon 35358, Republic of Korea; yellowtb@naver.com (M.J.); ssyang74@cnu.ac.kr (S.-s.Y.); 2 Department of Rehabilitation Medicine, Chungnam National University Hospital, Chungnam National University College of Medicine, Daejeon 35015, Republic of Korea; seralla@cnuh.co.kr

**Keywords:** Leigh syndrome, MT-ATP6, mitochondria, global developmental delay, feeding difficulties, torticollis

## Abstract

**Highlights:**

**What are the main findings?**

- An infant referred for suspected congenital torticollis was ultimately diagnosed with MT-ATP6-related Leigh syndrome.
- The case demonstrated subtle systemic features—feeding difficulties, recurrent vomiting, and failure to thrive—rather than early severe neurological symptoms.

**What are the implications of the main findings?**

- Early application of standardized infant assessment tools, including movement assessment and structured feeding evaluation, can detect subtle neurological dysfunction before overt symptoms appear.
- Careful follow-up with neuroimaging and genomic testing is essential for timely recognition and management of Leigh syndrome.

**Abstract:**

Leigh syndrome is a rare, progressive mitochondrial disorder of childhood. Early diagnosis is often challenging due to nonspecific clinical manifestations. We report a 1-month-old male infant initially referred for suspected congenital muscular torticollis who ultimately received a diagnosis of Leigh syndrome. Despite unremarkable perinatal history, he subsequently developed persistent feeding difficulties, recurrent vomiting, failure to thrive, and global developmental delay. Early neurological assessment revealed poor repertoire patterns on General Movement Assessment. The Neonatal Oral-Motor Assessment Scale (NOMAS) demonstrated dysfunctional oral-motor control, whereas the video fluoroscopic swallowing study (VFSS) revealed aspiration during swallowing. Brain MRI revealed symmetric basal ganglia lesions. Expanded whole-exome sequencing identified a pathogenic MT-ATP6 m.8993T>G variant with high heteroplasmy level (>90% in blood), confirming the diagnosis of Leigh syndrome. The variant was maternally inherited, although neither the mother nor the older sibling exhibited clinical features of mitochondrial disease. Leigh syndrome can initially manifest with subtle systemic features rather than overt neurological features. Persistent feeding difficulties and growth delay in infancy warrant thorough evaluation, including neuroimaging and comprehensive genomic testing, to enable timely diagnosis and optimize clinical management.

## 1. Introduction

Congenital muscular torticollis is a relatively common musculoskeletal condition in infants, typically presenting with head tilt and limited cervical motion. Most cases are benign and respond favorably to conservative management [1]. However, when associated with systemic symptoms such as feeding difficulties, developmental delay, or failure to thrive, alternative diagnoses must be considered. Mitochondrial disorders, including Leigh syndrome, are rare but clinically significant causes of neurodevelopmental impairment. We present a case of an infant initially evaluated for suspected torticollis who was ultimately diagnosed with Leigh syndrome.

Leigh syndrome, also known as subacute necrotizing encephalomyelopathy, represents one of the most common pediatric presentations of mitochondrial disease, with an estimated incidence of 1:40,000 live births [2,3]. This progressive childhood-onset neurodegenerative disorder results from defects in mitochondrial energy metabolism, and primarily affects organs with high metabolic demands including the brain, heart, and skeletal muscle. Leigh syndrome primarily affects oxidative phosphorylation and ATP synthesis caused by either mutations in mitochondrial DNA (mt DNA) or nuclear DNA [4]. The MT-ATP6 gene, encoding a subunit of mitochondrial complex V, represents one of the most frequently mutated genes in Leigh syndrome [5]. Clinical manifestations include developmental delay, initial generalized hypotonia, muscle weakness, progressive dystonia, swallowing difficulties, recurrent vomiting, respiratory abnormalities, seizures, or visual impairment. Brain magnetic resonance imaging (MRI) characteristically demonstrated symmetric lesions in the basal ganglia, thalamus, brainstem, or cerebellum [6,7].

Despite advances in diagnostic techniques, the early recognition of Leigh syndrome remains challenging due to nonspecific initial presentations that may mimic more common pediatric conditions. Recent developments in infant neurological assessment, including standardized evaluation tools such as Prechtl’s General Movement Assessment and the Neonatal Oral-Motor Assessment Scale (NOMAS), have enhanced clinicians’ ability to detect subtle neurological abnormalities in early infancy that may herald underlying neurodevelopmental disorders [8,9].

We present an instructive case of an infant initially evaluated for suspected congenital torticollis who ultimately received a diagnosis of MT-ATP6-related Leigh syndrome. This case demonstrates how systematic application of infant neurological assessment tools can facilitate early detection of neurological dysfunction, highlighting critical diagnostic decision points and clinical learning opportunities that can inform pediatric practice.

## 2. Case Presentation

A 1-month-old male infant was referred for the evaluation and management of suspected torticollis. The patient was born at 39 weeks of gestation via normal spontaneous vaginal delivery, with a birth weight of 2.94 kg. The perinatal course was unremarkable. Standard newborn screening performed through the Korean national program yielded normal results. The primary concern at presentation was intermittent head tilting to the left side, which had become more pronounced since birth according to maternal report. Physical examination revealed full passive range of motion of the neck without palpable sternocleidomastoid masses. Head tilting was mild and inconsistent, and no plagiocephaly was present. Primitive reflexes including Moro and Galant responses were intact, and muscle tone appeared normal. However, assessment of general movements using Prechtl’s method revealed a poor repertoire pattern, characterized by monotonous movement sequences with reduced complexity and limited variation in speed and amplitude of limb movements. General movements were first assessed in person by a certified assessor (JYC) who had completed the General Movements Advanced Course. Ultrasonography of the sternocleidomastoid muscles demonstrated no mass-like lesions or structural abnormalities. During the initial assessment, the mother reported significant feeding challenges that had persisted since birth. The infant consumed approximately 500–600 mL of formula daily, but each 100 mL feeding required nearly 40 min to complete and was frequently accompanied by vomiting. At one month of age, the patient’s height, body weight, and head circumference corresponded to the 45th (Z = −0.1), 32nd (Z = −0.5), and 81st (Z = +0.9) percentiles, suggesting growth parameters within the normal range for age.

At the 3-month follow-up visit, feeding difficulties persisted despite multiple compensatory strategies including paced feeding techniques and chin support during feeding. Despite these interventions, approximately 100 mL of formula continued to require more than 30 min per feed. A certified clinician conducted the feeding observation using the Neonatal Oral-Motor Assessment Scale (NOMAS), which revealed reduced jaw excursion and a clenching pattern consistent with a dysfunctional swallowing pattern. The prolonged feeding duration and recurrent vomiting prompted further evaluation with video fluoroscopic swallowing study (VFSS). This assessment demonstrated repeated aspiration events, yielding a Penetration-Aspiration Scale score of 7 indicating that material enters the airway, passes below the vocal folds, and is not ejected from the trachea despite effort. Neurological examination revealed concerning developmental findings including absent neck control, ataxic limb movements, and inability to sustain prone positioning during tummy time. By four months of age, the patient’s height, body weight, and head circumference had declined to the 11th, 0.8th, and 8th percentiles, respectively, reflecting marked growth faltering relative to the previous assessment. These findings, combined with persistent failure to thrive and feeding dysfunction, indicated the need for comprehensive neurological evaluation.

**Figure 1:**
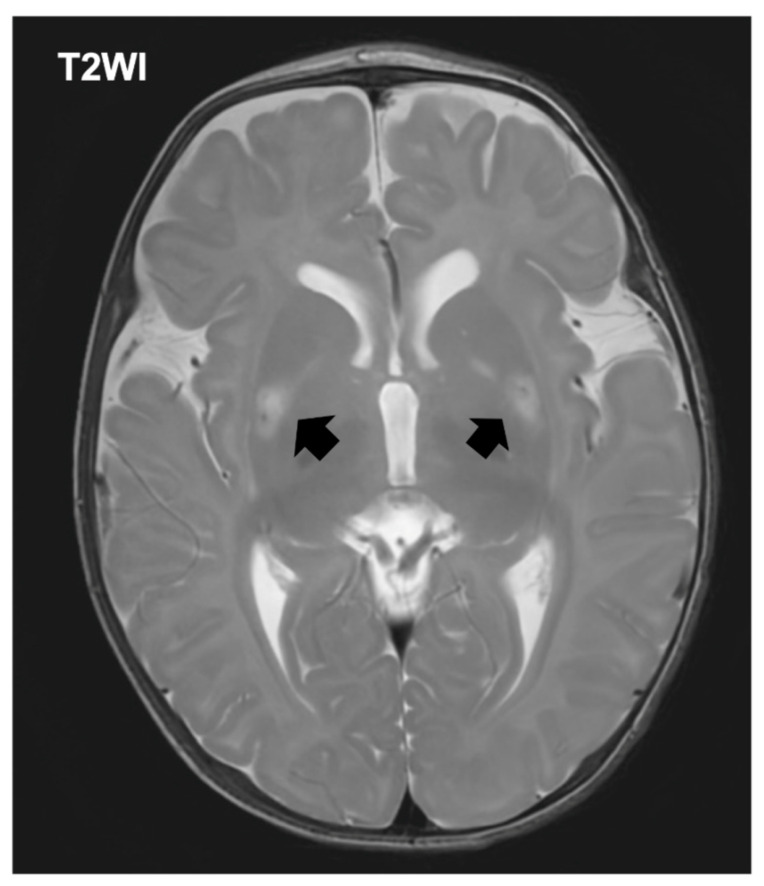
Axial T2-weighted brain MRI at 4 months of age demonstrating bilateral basal ganglia hyperintensities (arrows).

The patient was referred to a tertiary pediatric hospital for specialized evaluation including neuroimaging and genetic testing. The initial laboratory evaluation demonstrated hyperlactatemia with a plasma lactate level of 4.0 mmol/L (normal range [NR]: 0.5–1.6), accompanied by an elevated pyruvate concentration of 2.63 mmol/L (NR: 0.03–0.10). Creatine kinase was within reference limits at 63 U/L (NR: 34–204). Notably, ammonia was below the institutional reference interval at <9 μmol/L (NR: 11–51). Brain MRI revealed symmetric signal abnormalities in the basal ganglia bilaterally, consistent with the characteristic pattern observed in Leigh syndrome (Figure 1). Expanded exome sequencing using a hybrid nuclear exome and mitochondrial DNA panel identified a pathogenic variant in the MT-ATP6 gene (m.8993T>G), confirming the diagnosis of MT-ATP6-related mitochondrial disease manifesting as Leigh syndrome (OMIM:551500). The mutation load in blood was determined to be greater than 90%, consistent with the severe phenotype observed (Table S1).

Parental genetic testing confirmed maternal inheritance of the identified variant. Comprehensive family history revealed no reported hereditary disorders, and neither the mother nor the patient’s older sibling exhibited clinical features suggestive of mitochondrial disease. The asymptomatic status of family members likely reflects the variable expressivity and incomplete penetrance characteristic of mitochondrial disorders, possibly related to differences in mutation load across tissues and individuals

At the 6-month follow-up, feeding difficulties persisted and required nasogastric tube supplementation. NG supplementation was commenced at 6 months, prompted by a decline in daily oral intake to <400 mL and failure to thrive, and was continued through the 6-month assessment; developmental progress remained markedly delayed across all domains, consistent with severe early-onset Leigh syndrome.

## 3. Discussion

This case illustrates several critical aspects of pediatric mitochondrial disease diagnosis and highlights important clinical learning points for practitioners evaluating infants with apparent musculoskeletal concerns. The patient’s initial asymmetric head posture appropriately prompted consideration of congenital muscular torticollis, a common and typically benign condition in early infancy. Reports of intermittent head tilt and positional preference justified focused assessment of cervical alignment, range of motion, and cranial shape. However, the absence of sternocleidomastoid mass and preserved cervical mobility lowered the likelihood of classic muscular torticollis. The coexistence of persistent feeding difficulty raised early concern for non-mechanical pathology. As outlined in the diagnostic timeline (Table 1), the persistence of these red-flag features—failure to thrive and developmental delay—necessitated broadening the differential diagnosis to include neurological etiologies. This diagnostic evolution highlights the importance of systematic re-evaluation when common conditions present with atypical or incongruent findings.

The application of standardized infant neurological assessment tools proved instrumental in the early detection of abnormalities in our patients. The poor repertoire pattern identified through Prechtl’s General Movement Assessment, characterized by monotonous movement sequences and reduced complexity, provided an early objective indicator of neurological dysfunction despite the infant being born at term [8]. Feeding observation using the NOMAS revealed reduced jaw excursion with a clenching pattern consistent with a dysfunctional swallowing pattern. This NOMAS dysfunction pattern is associated with neurological impairment and suggested the need for comprehensive neurological evaluation beyond the initial musculoskeletal assessment [9]. The integration of standardized neurological assessment tools in routine infant evaluation can significantly enhance early detection of subtle neurological abnormalities that may indicate underlying neurodevelopmental disorders.

This case highlights important distinctions from previously reported MT-ATP6-associated Leigh syndrome cases. The MT-ATP6 gene, encoding a subunit of mitochondrial complex V, represents one of the most frequently mutated genes in Leigh syndrome [2,3,5,6]. Akar et al. described a patient with this variant who manifested severe neurological impairment from birth, profound lactic acidosis, and multiorgan failure [2]. In contrast, our patient was initially referred with suspected congenital torticollis and did not show overt neurological symptoms such as seizures or encephalopathy at presentation. Instead, the clinical course was characterized by subtle yet persistent systemic features, including prolonged feeding difficulties, recurrent vomiting, failure to thrive, and global developmental delay. Through this case, we confirmed that accurate neurological assessment during the neonatal period is inherently difficult, and in infants without distinctive neurological manifestations such as seizures, it is crucial not to overlook subtle findings obtained through maternal history. Continuous follow-up and careful clinical observation are essential for reaching an accurate diagnosis. It is also important to note that vomiting is a common symptom in infancy and may often be misinterpreted as a benign gastrointestinal problem [10]. Feeding difficulty and vomiting in early infancy are frequently attributed to benign gastrointestinal or airway conditions—such as physiologic gastroesophageal reflux, cow’s milk protein allergy, hypertrophic pyloric stenosis, eosinophilic esophagitis, laryngomalacia, or oral-motor delay—and may therefore be managed conservatively without further etiologic clarification. When vomiting is accompanied by failure to thrive and persistent feeding difficulties, a comprehensive evaluation is critical to ensure accurate diagnosis of underlying metabolic diseases such as Leigh syndrome. Notably, while feeding difficulty can occur in Leigh syndrome, primary feeding-led presentation at the initial encounter is uncommon. In a large multicenter cohort study, feeding or sucking difficulties were the presenting feature in only 14.1% of patients, compared to abnormal motor findings in 82.8% [11]. This relative rarity of feeding-predominant presentation may delay consideration of mitochondrial etiologies when overt focal neurologic signs are absent and newborn screening is normal at birth.

The mitochondrial 8993T>G variant accounts for approximately 10–15% of all Leigh syndrome cases and was first identified by Tatuch et al. in 1992 as a cause of maternally inherited disease [12]. Recent studies have established important genotype-phenotype correlations, with the m.8993T>G variant typically associated with severe early-onset disease and characteristic biochemical abnormalities including elevated lactate and reduced plasma citrulline levels [5]. In the past, mitochondrial DNA analysis had to be performed separately from nuclear gene testing. However, the use of an expanded exome sequencing approach with a combined nuclear exome and mitochondrial genome hybrid capture panel now enables the simultaneous detection of nuclear and mtDNA variants, thereby enhancing diagnostic efficiency in genetically heterogeneous disorders such as Leigh syndrome [4,13].

Although parental testing confirmed maternal inheritance of the MT-ATP6 m.8993T>G variant, both the mother and the sibling were clinically asymptomatic. This finding can be explained by the heteroplasmic nature of mitochondrial DNA mutations, in which phenotypic expression depends on tissue-specific mutant load and threshold effects [14]. Heteroplasmy levels can vary widely even among related individuals, leading to phenotypic variability despite shared mutations. In this case, the maternal heteroplasmy quantification was not performed, as the family declined additional testing after variant inheritance was confirmed, limiting the precision of recurrence risk counseling. We therefore discussed reproductive options (PGT-M, prenatal diagnosis with counseling on tissue mosaicism) and agreed on a stepwise plan for the asymptomatic female sibling, prioritizing developmental/growth surveillance and symptom-triggered testing, with genetic testing scheduled after counseling to align with family preferences and ethical considerations.

Early recognition of Leigh syndrome enables not only appropriate counseling but also timely initiation of supportive and targeted interventions, including mitochondrial cofactors, nutritional optimization, and respiratory management. Early diagnosis also allows anticipatory monitoring for complications such as metabolic decompensation or feeding failure, and facilitates family-centered counseling and reproductive planning, which together contribute to improved quality of life and long-term outcomes.

## 4. Conclusions

This case demonstrates that Leigh syndrome can initially manifest with subtle systemic features rather than overt neurological impairment in early infancy. The diagnostic journey from suspected congenital torticollis to confirmed mitochondrial disease emphasizes several key clinical principles: persistent feeding difficulties and growth delay in infants warrant comprehensive evaluation; systematic neurological assessment including imaging and genetic testing is essential when red flag symptoms are present; and early recognition of mitochondrial disease enables appropriate family counseling and supportive management strategies. The evolution of diagnostic techniques, particularly next-generation sequencing, has enhanced ability to identify rare disorders that may initially mimic more common conditions. Continued awareness of these diagnostic challenges and systematic approach to evaluation remain essential for optimizing outcomes in infants with complex presentations.

## Figures and Tables

**Table 1:** Presentation of the diagnostic timeline and decision-making process.

Age	Key Clinical Event	Assessment Performed	Findings	Impact on Decision-Making
1 month	- Intermittent left head tilt - Feeding difficulty	- Physical exam (Neck ROM, Prechtl’s method) - Ultrasonography of neck muscles	- Full passive ROM, No sternoclavicular muscle mass - General movements: poor repertoire	- Classic congenital muscular torticollis less likely - Planned structured feeding assessment
3 months	- Persistent feeding difficulty and vomiting - Developmental delays	- NOMAS - VFSS - Neurological examination	- NOMAS: reduced jaw excursion/clenching (dysfunctional pattern) - VFSS: PAS 7 with repeated aspiration - Absent neck control, ataxic limb movements	- Referred to tertiary pediatric hospital for comprehensive neurological evaluation
4 months	- Etiologic work-up	- Biochemical laboratory test - Brain MRI	- Hyperlactatemia - Symmetric bilateral basal ganglia hyperintensities	- Suspected Leigh syndrome
5 months	- Genetic confirmation	- Whole exome sequencing (WES)	- Pathogenic MT-ATP6 m.8993T>G variant - mtDNA heteroplasmy detected	- Diagnosis: MT-ATP6-related Leigh syndrome - Initiated counseling and supportive care plan
6 months	- Clinical course	- Follow-up	- Pathogenic variant; >90% blood heteroplasmy - Persistent feeding difficulty and a decline in daily oral intake → NG supplementation	- Ongoing multidisciplinary management

ROM, range of motion; NOMAS, Neonatal Oral-Motor Assessment Scale; PAS, penetration aspiration scale.

## Data Availability

The original contributions presented in this study are included in the article/Supplementary Material. Further inquiries can be directed to the corresponding author.

## Supplementary Materials

The following supporting information can be downloaded at: https://www.mdpi.com/article/10.3390/children12111522/s1, Table S1: Summary of Molecular Findings in the Present Case.
