# Supplementary material for: From Congenital Torticollis to Leigh Syndrome: A Case Report of Diagnostic Evolution in an Infant
Source: Children (Basel). 2025 Nov 10;12(11):1522. doi: 10.3390/children12111522 (PMC12651586; doi:10.3390/children12111522)
Supplement: Supplementary file 1 [file children-12-01522-s001.zip › children-3928763-supplementary.pdf]

Supplementary Table S1. Summary of Molecular Findings in the Present Case

Category	Description	Findings	Reference/Remarks
Gene	Mitochondrial ATP synthase subunit 6 (MT-ATP6)	MT-ATP6	Encodes a subunit of mitochondrial complex V
Nucleotide change	Single base substitution in mtDNA	m.8993T>G	Reported pathogenic variant
Amino acid change	Missense mutation	p.Leu156Arg	Alters ATP synthase subunit structure
Genomic location	Mitochondrial genome, position 8993 (NC_012920.1)	—	Common hotspot region for Leigh/MILS phenotypes
Variant type	Point mutation (transversion)	T → G at nucleotide 8993	—
Heteroplasmy level	Mutant load in blood sample	>90% (blood)	Associated with severe early-onset phenotype
Inheritance pattern	Maternal	Variant detected in maternal mtDNA	Mother clinically unaffected
